# Commodification of Transitioning Ethnic Enclaves

**DOI:** 10.3390/bs4040341

**Published:** 2014-09-29

**Authors:** Kathryn Terzano

**Affiliations:** Department of Geography and Regional Planning, Westfield State University, 577 Western Avenue, Westfield, MA 01086, USA; E-Mail: kterzano@westfield.ma.edu; Tel.: +1-413-572-8314; Fax: +1-413-572-5470

**Keywords:** ethnic enclaves, branding, commodification

## Abstract

This literature review examines the changing roles of ethnic enclaves, the question of their authenticity, and their value as commodified spaces, giving special attention to Little Italy neighborhoods in the United States. Understanding the roles of ethnic enclaves requires some understanding about immigrants’ identities. For some theorists, immigrants become blended into society over the course of generations; for other theorists, descendants of immigrants sometimes retain their cultural heritage and traits, helping form a multicultural or pluralist society. In the traditional sense, ethnic enclaves consist of both ethnic residents and ethnic businesses (such as restaurants, shops, and grocers). One way that ethnic enclaves change is when the area experiences a demographic shift, and people from outside the ethnic group move their residences and businesses to the neighborhood, resulting in the area becoming diversified in people and businesses. A second way that an ethnic enclave changes is when the ethnic group shrinks, but the shops and other businesses remain, resulting in the area becoming diversified in residents but not businesses. This latter situation may encourage commodification of the neighborhood’s ethnic identity, where a municipality or business association seeks to preserve an enclave’s ethnic reputation for tourism purposes. This commodification has implications for many individuals and groups within the enclave as well as outside of it.

## 1. Traditional Role of Enclaves

Prior to the late 19th Century, ethnic enclaves in North America were seen as temporary places as immigrants assimilated into the host society [1]. Classic assimilation theorists were concerned with how immigrants adapted over time to fit into American society and, furthermore, these theorists saw it as necessary—And inevitable—For immigrants to eventually shed their ethnic identities to become American themselves [1,2]. Regardless of whether one agrees with classic assimilation theory, it is helpful to look at these theorists’ work because they studied how immigrant communities are formed and the purposes that ethnic enclaves have served. As immigrants arrived in the U.S. and Canada, they settled in areas where their compatriots lived. This process, referred to as chain migration, gave newcomers easier access to housing, jobs, and socializing, particularly when they spoke little or no English. As the number of immigrants grew, communities rose up around them. In enclaves, immigrants could find familiar culture–grocers that sold ethnic food; churches and schools where the immigrants’ native language was spoken; and businesses that were based on skills and goods for which the immigrants were well known. In their new country, immigrants recreated the amenities of their former countries and in doing so, created communities [1,2,3,4]. This trend of ethnic enclaves providing a landing pad for recent arrivals continues today—What has changed is the ethnicity of the groups creating new ethnic enclaves; rather than new Little Italies being formed, today’s newly created enclaves are created by recently arrived immigrants from Southern and Southeast Asia, the Caribbean, and Latin America [1].

## 2. Changing Enclaves

### 2.1. Demographic Changes

Most research on ethnic enclaves discusses either the formation of the enclave or the economic benefits to members of it in the form of ethnic enclave economies [3]. Little attention has been paid to the dissolution of enclaves, and although the Chicago School model would explain that acculturation has occurred, this is not always accurate since straight-line assimilation into the American mainstream is not the only explanation for why enclaves fade away [3]. Alternately, some ethnic enclaves were destroyed during the 1950s through to the 1970s, when urban renewal was a common practice in city planning departments and one result of this policy was the destruction of many minority neighborhoods [5]. For example, Boston had a thriving, working-class, Italian-American neighborhood in its West End, which was destroyed by urban renewal in the 1960s, displacing the residents who had lived there [6].

In the traditional sense, ethnic enclaves consist of both ethnic residents and ethnic businesses (such as restaurants, shops, and grocers). One way that ethnic enclaves change is when the area experiences a demographic shift, and people from outside the ethnic group move their residences and businesses to the neighborhood, resulting in the area becoming diversified in people and businesses. One notable study looked at this kind of gentrification in Little Portugal, Toronto [7], where non-Portuguese-Canadians are moving in and displacing the Portuguese population. In this particular neighborhood, demographic change has resulted from Portuguese and Portuguese-Canadian residents moving out of the neighborhood combined with an in-movement of new arrivals from Africa, Asia, and Latin America [7].

A second way that an ethnic enclave changes is when the ethnic group shrinks, but the shops and other businesses remain, resulting in the area becoming diversified in residents but not businesses. In this situation, the ethnic group may have shrunk because members have died off, moved away, or some combination of the two. This situation may encourage commodification and branding of the neighborhood’s ethnic identity because this ethnic identity may give the neighborhood coherency and may be widely regarded by city officials, neighborhood residents, and area business owners as a drawing factor for local tourism.

The emergence of a new kind of ethnic enclave, the so-called ethnoburb, because of its location in the suburbs of cities, has attracted some attention [8]. In certain instances, ethnoburbs arise because of new immigrants to the area, but in other cases ethnoburbs form when urban residents move from existing ethnic enclaves to these suburban locations [8]. In this sense, the emergence of ethnoburbs can be seen as related to the dissolution of urban ethnic enclaves. The development of ethnoburbs relates to the overall trend of suburbanization in North America, and has been observed among multiple ethnic groups and in various cities [7,8,9]. These ethnoburbs may represent the future of ethnic enclaves, especially in cities where low-rent properties are increasingly rare.

#### Enclaves and Changing Ethnicities

Occasionally, instead of the neighborhood becoming more diverse in its population and businesses, a second minority ethnic group displaces the original ethnic group, and the enclave transitions into a different ethnic enclave. As an example, Little Italy in Manhattan has contracted over the years as Italians and Italian-Americans have moved out and neighboring Chinatown has increasingly expanded into Little Italy [10]. The result is that some streets that have been part of Little Italy, historically, are now culturally affiliated with Chinatown [10].

In Little Portugal, Toronto, the pattern of one ethnic group being replaced by a subsequent, second ethnic group is related to suburbanization and gentrification [7]. Other ethnic groups, such as Jewish and Italian groups, have increasingly moved to the suburbs, but the Portuguese relocation to the suburbs has coincided with this area of Toronto (West-central Toronto) experiencing increased gentrification since the 1980s [7]. Murdie and Teixeira [7] cover this topic of gentrification of ethnic enclaves extensively in their article, using key informants and focus groups to report on differences of perceptions of gentrification among Portuguese (including Portuguese-Canadians), non-Portuguese recent immigrants, and British (including British-Canadians). Murdie and Teixeira’s study [7] treats new residents as a group (“gentrifiers”) that is displacing the Portuguese-ethnic population rather than diversifying the population of the neighborhood. Although Murdie and Teixeira discuss certain positive impacts from gentrification, such as the potential for stabilization of a declining area, the emphasis of the findings is on the negative impacts of gentrification [7].

Other ethnic enclaves appear to experience coethnicity, or what Luk and Phan [11] argue are multiple ethnic groups coexisting within a single ethnic enclave, leading to an enclave that is an amalgam of two or more ethnicities. In their case study of Chinatown West, they focus on the businesses in the neighborhood and document the increasing presence of Sino-Vietnamese businesses [11]. They discuss how these Sino-Vietnamese businesses are replacing the departing Hong Kong Chinese businesses, and they characterize this change as a transition, but they also argue for the recognition that one enclave can have multiple ethnicities—As opposed to defining an ethnic enclave as being composed of one ethnic group only [11]. This line of thinking is in agreement with earlier work by Harney [12], who finds that ethnic enclaves often have more than one ethnic group present, as well as with Wei [8], who finds that suburban ethnic enclaves are generally multiethnic. However, this conflicts with at least one recent study [13]. In this study, Zhou offers a case study of Koreatown in Los Angeles—Where, at the time of writing, Latinos (mainly Mexicans, Salvadorans, and Guatemalans) comprised about two-thirds of the population and Koreans comprised only about one-fifth of the population—But notes that such a multiethnic enclave is unusual [13].

### 2.2. Commodification and Branding

When members of an ethnic group move out of the enclave or their numbers within the enclave thin, but the municipality or others decide to preserve the enclave’s ethnic reputation for tourism, the ethnic identity has been turned into a commodity. Sometimes this commodification is evident through physical identifiers in the enclaves, such as banners that advertise the enclave’s name, posters, and other forms of traditional advertisement [14].

Consider Chinatown in Washington, D.C. It is no longer where a majority of ethnic Chinese in the District lives; yet, the city uses the marketing of Chinese restaurants and ethno-cultural diversity for tourism dollars [15]. The area is artificially preserved through the actions of city planners and a handful of Chinatown business owners, who began their efforts to reinvigorate Chinatown after the neighborhood experienced population loss following the race riots of the 1960s. For example, Washington, D.C.’s Office of Planning has design review criteria for new businesses in Chinatown that include the “contribution of building design, including signage and awnings, to the Chinese identity of Chinatown” [16]. These shops and restaurants attract tourists who value the cultural or ethnic other, and whose spending offers support for the preservation of the area, as well as generating revenue through the city [15]. Tourists, in this sense, refer to both local residents who live outside the enclave as well as people visiting from other cities. A similar argument that commodification is taking place could be made for Koreatown in Los Angeles, whose Korean population, as noted above, is smaller than its Latino population, yet the neighborhood continues to be branded as Koreatown [13].

Occasionally, this ethnic identity is perpetuated by outsiders as well. In the case of Little Italy, New York, a peculiar phenomenon exists where Albanian Kosovars pass themselves off as Italian [17]. These Albanian Kosovars largely work in the restaurant industry and market both their own ethnic identity, as well as the restaurants’ associated ethnicity, as being Italian, even adopting Italian and Italian-American nicknames and using common Italian phrases, such as for greetings [17]. To outsiders, such as tourists to Little Italy, these Albanian Kosovars appear to be Italians.

#### 2.2.1. The Role of Enclaves for Outsiders

Mainstream America has held various views of ethnic enclaves over the years. Prior to around World War II, enclaves were seen as necessary to keep ethnic and non-white individuals separated from white Americans [1]. For some ethnic groups, such as northwestern European groups, enclaves developed wherever the groups chose to settle with few restrictions. For groups seen as non-white (including Jews and Italians, historically), geographical choices were more limited, especially on the city level, as spatial discrimination through practices such as redlining kept racial and ethnic groups separated [1,18,19]. These non-white enclaves, particularly Asian enclaves, came to be seen as exotic with the hint of danger, crime, and illicit activities (e.g., prostitution, opium dens), which simultaneously repelled and intrigued white America [1,20].

Although everyday residents of enclaves have generally been unsupportive of marketing efforts toward outsiders, business owners in enclaves realized long ago they could make a greater profit by attracting these outsiders to their businesses [20,21]. Rodriguez notes how, during the 1930s, business owners in Chinatown, San Francisco, supported installing “Chinese-style storefronts”, and some Chinatown merchants required their shop employees to wear traditional Chinese clothing during festivals [20] (p. 72). These decisions were made as part of a larger marketing strategy to attract non-Chinese tourists to the neighborhood.

Umbach and Wishnoff specifically address the split between the merchant elites and the everyday residents of Manhattan’s Chinatown. They discuss three city planning initiatives between 1950 and 2005 that, they argue, were attempts to “fashion an urban space that exploited exoticized notions of the quarter and its residents” [21] (p. 214). However, while the everyday residents may have resisted these initiatives, the merchants were in favor of these schemes going forward. Furthermore, each of these planning schemes involved physical changes to Chinatown to highlight the Chinese ethnic character of the neighborhood. Umbach and Wishnoff [21] refer to these physical changes as “faux-Chinese architecture” throughout their article, and describe how this kind of exoticized architecture was thought to attract tourists, whose spending benefits the merchants. Umbach and Wishnoff [21] are careful to make the distinction between the merchants and everyday residents. They report on how the everyday residents generally opposed the three planning schemes. For example, one of the planning schemes proposed installing a Chinese-style arch in 2005, but the residents opposed construction that appeared to be directed to tourists and not Chinatown residents.

Mazumdar *et al.* [22] discuss a different perspective through their examination of Little Saigon in Westminster, California (which is in the Los Angeles area). In their study of this enclave, they found via interviews that residents supported having Vietnamese-style architecture in their neighborhood. Whereas residents in Rodriguez’s San Francisco Chinatown and Umbach and Wishnoff’s Manhattan Chinatown found the Chinese-style architecture to be inauthentic, this was not the case in Little Saigon. Instead, Mazumdar *et al.* [22] see the physical construction of ethnic identity to be an important component alongside the social construction and maintenance of Vietnamese identity within the enclave. They argue that Little Saigon’s Vietnamese-style architecture “communicates its Asian heritage, reaffirming ethnic identity, expressing nostalgia for places left behind, and ‘engraving’ on the new landscape memories from the past” [22], (p. 323). They further contend that the Vietnamese-style architecture announces the Vietnamese community to outsiders, serving as a welcome form of marketing about the goods and services available within the neighborhood.

#### 2.2.2. The Question of Authenticity

Several studies have examined the role of ethnic enclaves for outsiders—Their commodification of the ethnic identity for business or tourism purposes—And have connected this concern with whether these neighborhoods, when commodified, are authentically ethnic [6,7,9,15,20,21,23]. When ethnic enclaves retain their residential component, they may also be seen as retaining their ethnic identity in an authentic way. For example, Hackworth and Rekers [9] discuss four Toronto neighborhoods that are also Business Improvement Areas. Each of these neighborhoods is branded as an ethnic enclave, having a strong commercial ethnic identity associated with it, and elite merchants and the city have purposefully maintained these commercial ethnic identities to increase tourism dollars [9]. Thus, it is the commercial identity that attracts tourists, not the residential identity.

Hackworth and Rekers’ research attempts to describe markers of authenticity in an ethnically identified neighborhood. One marker concerns the residential component of the neighborhood. If the residents of the neighborhood identify with the same ethnicity as that of the neighborhood identity, the neighborhood is thought by the authors to be more authentically ethnic. Additionally, if residential real estate in the neighborhood is comparably priced to other neighborhoods in the metropolitan area, this is another sign of authenticity. Hackworth and Rekers differentiate between two commercially Italian-ethnic neighborhoods in Toronto, one of which maintains an Italian residential component and is thought of as authentic, whereas the other neighborhood is only commercially Italian and is thought to be less authentic.

Hackworth and Rekers [9] argue that an increase in the number of restaurants and a decrease in the number of grocers is another sign of an ethnic neighborhood that is catering more to tourists than its own residents. In turn, a neighborhood that focuses on attracting tourists more than serving its own residents is thought to be inauthentic as an ethnic enclave. Hackworth and Rekers go as far as to claim, “Ethnically labeled BIAs (Business Improvement Areas) package and reproduce ethnicity for consumption, primarily for tourists and nonresident ethnics in the region.” [9], (pp. 231–232). Thus, having organizations in place to manage ethnic enclaves, they argue, is partially what is leading to the commodification of these same enclaves.

Performances of an ethnic identity, such as Albanian Kosovars using Italian nicknames, or the wearing of traditional Chinese clothing to help create a desirable ambiance for a shop in Chinatown, may call into question whether these are authentic spaces [17,21]. Sharon Zukin [24] argues authoritatively that although the concept of authenticity can be contentious since cities are dynamic places, there is a loss of authenticity as immigrant neighborhoods experience gentrification. While an explicit discussion of authenticity is notably absent from Murdie and Teixeira’s study on Little Portugal, Toronto, the authors discuss how without a continued influx of Portuguese-ethnic residents, the neighborhood will cease to be a “complete enclave”, even if a commercial axis continues to thrive [7] (p. 79).

Furthermore, authenticity may be more accurately described as a matter of subjective perception, where the authenticity of any given neighborhood, or even a single store, may be perceived differently among different groups or individuals [24]. McClinchey [25] mirrors this argument, asserting that relative authenticity allows outsiders to have their own interpretations of whether a place is authentic. Even when a place is ostensibly fake (or inauthentic) through performances of a culture, such as, again, when 1930s San Francisco Chinatown employees were made by their employers to wear Chinese-style clothing, visitors may experience existential authenticity [20,23,25]. This concept of existential authenticity began as a philosophical one, originated by Heidegger to explain how individuals form meaning through experiencing and simply *being* (existing) [23,26,27]. Thus, individuals may visit a store or watch a parade in an ethnic enclave and have experiences that are authentic for themselves, and perhaps shared among their group (termed intragroup existential authenticity), regardless of whether the culture being enacted would be considered authentic by others.

Existential authenticity, in turn, relates to ethnicity as a social construction, which is to say that society creates an understanding of what ethnicity means, rather than ethnicity having a fixed meaning [28,29,30]. By extension, how ethnicity continues to be created and recreated, enacted in settings or through events, and interpreted as part of one’s own self-identity, is very much a subjective experience. The potential differences in interpretation of authenticity may lead to intragroup and intergroup conflicts when some stakeholders perceive a place to losing its authenticity while other stakeholders celebrate the marketing of the neighborhood.

#### 2.2.3. Commodification in Italian Ethnic Enclaves

Commodification has also happened with Italian ethnic enclaves, in addition to the example already given above of Little Italy, New York [9,31]. In Little Italy, Toronto, most of the Italian-ethnic residents moved to the suburbs and a different Italian-ethnic neighborhood (Corso Italia) by the 1980s. To a large extent, Portuguese, Chinese, and Vietnamese immigrants repopulated the neighborhood, constituting about half the population of the area by 2005 [9]. However, the city has bolstered the neighborhood’s Italian identity by investing in a streetscape improvement plan, including thematic street signs that feature the Italian flag. Even though few Italians or Italian-Canadians live there, the area remains home to popular Italian restaurants [9].

In Little Italy, Cleveland, prominent residents and business owners commissioned the Little Italy Redevelopment Corporation in 1994 to improve and preserve the neighborhood, partly in response to fears of losing the neighborhood’s homeowners to the suburbs. The goals of the Little Italy Master Plan are reminiscent of the commodification of ethnic neighborhoods [9,15]. The master plan sought to market the unique character, enhance the public space, and to promote ethnic diversity and attract consumers [32]. The fact that business owners have sought to reinvigorate Little Italy is similar to how enclaves in other cities have been marketed [33].

San Diego, California, offers another example. In the early 20th century, Italian fishermen and their families lived in an ethnic enclave, but this neighborhood was largely destroyed by 1962 with the completion of an urban renewal-era highway through the neighborhood [34,35]. The area continued to decline until the 1990s, when the city invested money into branding the area as Little Italy, which included installing a large, metal “Little Italy” street sign and building Amici Park [23]. New condominium buildings were given Italian-sounding names [35]. However, Kayzar calls the Italian theme of the neighborhood an “illusion”, citing how the area is predominantly commercially Italian while few residents are Italian or Italian American [23] (p. 135).

#### 2.2.4. Positive and Negative Impacts from Commodification

Cities can benefit from commercialized or commodified enclaves through increased tourism and taxes where local and out-of-town visitors to the enclave spend money at neighborhood businesses. An ethnic enclave may also serve as a cultural reservoir, preserving the history of the community through architecture, elements of the streetscape (such as sidewalk pavers), and the stores and restaurants themselves. Cultural festivals, a prominent feature of many ethnic enclaves, may also serve an important role in preserving the heritages and traditions, while simultaneously bringing in tourists [25].

Previous research has examined the motivations of the visitors or tourists as being an attraction to the cultural or ethnic “other” who visit enclaves to experience the food, language, customs, or overall culture of people different from themselves, and, thus, have their own existentially authentic experience [23,26,27,36,37]. Sometimes, social preservationists–outsiders who move to the enclave–encourage long-term residents to remain because those long-term residents add to the legitimacy of the enclave [38]. These long-term residents may also benefit from healthy profits on the sales of property, to the extent that commodification occurs alongside gentrification [7]. However, value might also exist for members of the associated ethnic group beyond the financial benefits from entrepreneurship or home sales. Even when the area is only commercially ethnic, it can still function as a place where members of an ethnic group can go to meet like members, buy specialty groceries or other goods, and, in some cases, converse in a non-English language. In the case of Chinatown in Washington, DC, the restaurants and shops form a node for Chinese people that they cannot find in a residential area [15,28].

Furthermore, an ethnic enclave may benefit those who have relatively weaker ties to their ethnicity identity. Gans coined the term symbolic ethnicity to describe “nostalgic allegiance to the culture of the immigrant generation, or that of the old country; a love for and a pride in a tradition that can be felt without having to be incorporated in everyday behavior” [39] (p. 9). He describes the grandchildren and great-grandchildren of immigrants as being largely disconnected from their ancestral country of origin, but, rather, enacting their ethnic identity symbolically [39]. This is situational ethnicity, where people voluntarily enact their ethnicity at the time and place of their choosing but can also choose to ignore their ethnicity [40]. An important distinction is that symbolic ethnicity is largely only available to those perceived as White; for others, ethnicity may be ascribed to them whether they desire the ethnic label or not. However, for an American grandchild of Italian immigrants, visiting an Italian ethnic enclave may have personal meaning, even if that person does not choose to live in Little Italy, eat Italian food any more frequently than other Americans, or marry another Italian American [39,40].

On the negative side, and as discussed above, the primary negative impact with commodification is the possible loss of authenticity or even a blurring of the lines between authentic and inauthentic [6,7,9,15,20,21,23,28]. A loss of authenticity represents a negative social or cultural impact, but negative economic impacts are also possible. Whereas commodification can help revitalize an economically struggling enclave through renewed interest in the area by entrepreneurs and visitors, some lower-income residents, including new immigrants, may be priced out of the neighborhood [7]. Additionally, the increased presence of tourists and visitors can exacerbate tensions between cultural groups, leading to intergroup conflict [7,40]. This may be particularly evident during festivals and parades held in the ethnic neighborhood, where members of the associated ethnic group may be affronted at the representation or depiction of their culture [25]. Lastly, some of the new entrepreneurs’ restaurants and other businesses may displace preexisting businesses–which again could lead to increased intergroup conflict–perhaps especially when the replacement businesses are purportedly but falsely and inauthentically ethnic [7,9,40].

### 2.3. Areas for Future Research

A longitudinal study following the changes in a specific enclave over a period of years or even decades would provide valuable information. A greater understanding of the responses of enclave residents and business owners would also be useful, including their perceptions of change, authenticity, and overall satisfaction with their neighborhood. Much of the existing literature on ethnic enclaves is in the form of cases studies employing surveys, focus groups and interviews, sometimes supplemented by demographic data from census records [7,8,9,11,15,17,21,22,38]. As always, a limitation with case study research is the inability to generalize the findings; a larger study of ethnic enclaves that does not rely on case studies would help provide breadth to the existing research. Finally, to the extent that further case studies are needed, a greater geographic diversity could be helpful, as enclaves in Toronto, Ontario, are already well represented, ostensibly because of the diverse population of the city and the prominence of its ethnic enclaves [3,7,9,11,12,25]. Case study research on an enclave in a city that has only a few ethnic enclaves may yield interesting differences from case studies on enclaves in cities that have many ethnic enclaves (e.g., Toronto, New York City).

## 3. Conclusions

When the ethnic group associated with an ethnic enclave diminishes over time, but the commercial aspect remains, city officials and business owners in the neighborhood may seek to commodify the neighborhood’s ethnic identity. This commodification has implications for many individuals and groups within the enclave as well as outside of it. These implications include both benefits and consequences, and in this way commodification can be similar to the experience of gentrification in other types of neighborhoods. However, given the sensitive nature of capitalizing on an ethnic identity and the potential interpersonal and intergroup conflicts that may arise from it, this issue is deserving of its own focus and would benefit from further study. 
